# Fluoroquinolones as the linchpin of tuberculosis therapy: contrasting efficacy and resistance profiles in *Mycobacterium tuberculosis* versus *Mycobacterium abscessus*

**DOI:** 10.3389/fcimb.2026.1833650

**Published:** 2026-05-28

**Authors:** Magdalena Kuzioła, Małgorzata Korycka-Machała, Daria Zygała-Pytlos, Bożena Dziadek, Malwina Kawka, Jakub Pawełczyk, Norbert Odolczyk, Piotr Zielenkiewicz, Marcin Słomka, Jarosław Dziadek

**Affiliations:** 1 Institute of Medical Biology, Polish Academy of Sciences, Łódź, Poland; 2 The Bio-Med-Chem Doctoral School of the University of Lodz and Lodz Institutes of the Polish Academy of Sciences, Łódź, Poland; 3 Department of Molecular Microbiology, University of Łódź, Faculty of Biology and Environmental Protection, Łódź, Poland; 4 Department of Bioinformatics, Institute of Biochemistry and Biophysics, Polish Academy of Sciences, Warsaw, Poland; 5 Faculty of Biology, University of Warsaw, Warsaw, Poland; 6 Faculty of Biology and Environmental Protection, University of Łódź, Centre for Digital Biology and Biomedical Science – Biobank Łódź, Łódź, Poland

**Keywords:** DNA gyrase, drug resistance, fluoroquinolones, Mycobacterium abscessus, Mycobacterium tuberculosis

## Abstract

**Introduction:**

Fluoroquinolones (FQs) are pharmacological cornerstone agents in global tuberculosis (TB) management, critical for both accelerating drug-sensitive regimens and defining the resistance spectrum of multidrug-resistant TB.

**Methods & Results:**

To evaluate the therapeutic potential of this class across the mycobacterial spectrum, we conducted a comprehensive phenotypic screening campaign utilizing OTAVA and ECBD pilot-libraries against reference strains of *Mycobacterium tuberculosis* (*Mtb*) and *Mycobacterium abscessus* (*Mab*). This initial screen, followed by detailed bactericidal concentration (BC) and cytotoxicity assessments, identified and selected potent FQ derivatives for in-depth characterization. These FQ compounds exhibited exceptionally high potency against *Mtb*, with several demonstrating robust bactericidal activity at concentrations far below toxic levels to mammalian cells. This activity was retained in clinically relevant, challenging states, as the compounds effectively eliminated *Mtb* residing within human macrophages and persisted within protective biofilm structures. In stark contrast, *Mab* displayed marked intrinsic resistance. Most FQ derivatives highly potent against *Mtb* required significantly higher concentrations for inhibition and bactericidal effect against *Mab*. For instance, a leading clinical FQ showed substantially diminished activity against *Mab* compared to *Mtb*. Analysis of acquired resistance mechanisms demonstrated that both *Mtb* and *Mab* primarily rely on similar acquired point mutations in the Quinolone Resistance Determining Regions (QRDRs) of the *gyrA* and *gyrB* genes. However, molecular docking indicated a slight but significant reduction in theoretical binding affinity to the *Mab* gyrase complex.

**Discussion:**

We hypothesize that the profound differential in intrinsic susceptibility is at least partially driven by non-target factors. This work supports the continued critical role of FQs in *Mtb* therapy while highlighting the importance of structural modifications that could address hypothesized intrinsic resistance mechanisms in *Mab*.

## 1 Introduction

Tuberculosis (TB), caused by *Mycobacterium tuberculosis*, remains a catastrophic global health crisis that demands continuous innovation in therapeutic and diagnostic strategies. Despite being a treatable and preventable disease, recent epidemiological data highlights a pervasive burden, with the total number of individuals falling ill with TB continuing to sustain high levels, reaching an estimated 10.7 million cases globally, even as global efforts have seen TB-related deaths slightly decline to approximately 1.23 million in 2024 (WHO, 2025). This sustained, high incidence underscores the necessity for shorter, more effective, and better-tolerated therapeutic regimens that can enhance patient adherence and reduce community transmission. Addressing the global prevalence and burden of multidrug-resistant tuberculosis has been the subject of extensive research (Zumla et al., 2014; Silva et al., 2018).

The most formidable challenge to global TB control is the escalating prevalence of drug-resistant TB (DR-TB), particularly multidrug-resistant (MDR-TB, defined by resistance to isoniazid and rifampicin) and rifampicin-resistant (RR-TB) strains. Since 2021, the efficacy of the fluoroquinolone (FQ) class, specifically moxifloxacin and levofloxacin, has become the decisive factor in classifying the severity of these resistance profiles (Alagna et al., 2021). MDR/RR-TB that is also resistant to any fluoroquinolone is now formally classified as Pre-Extensively Drug-Resistant TB (Pre-XDR-TB). Furthermore, Extensively Drug-Resistant TB (XDR-TB) is defined as MDR/RR-TB resistant to any fluoroquinolone and at least one additional Group A drug (bedaquiline or linezolid) (Alagna et al., 2021; Sinha et al., 2025). This formal classification confirms that the loss of FQ efficacy immediately moves a patient into the most difficult-to-treat categories, placing FQs at the critical threshold of therapeutic failure and highlighting their indispensable role as core Group A drugs in modern DR-TB management. The mechanism of action of FQs involves inhibition of key cellular processes, specifically DNA replication and transcription, and leads to impaired homologous recombination (Hooper, 2001; Bush et al., 2020).

The strategic inclusion of FQs is now central to global treatment guidelines, enabling the acceleration of therapy across the mycobacterial disease spectrum (Konstantynovska et al., 2025). For adults and adolescents (aged 12 years or older) with drug-susceptible pulmonary TB (DS-TB), the standard six-month course is replaced by a highly effective four-month regimen consisting of two months of isoniazid, rifapentine, pyrazinamide, and moxifloxacin, followed by two months of isoniazid, rifapentine, and moxifloxacin (2HPZM/2HPM) (Dorman et al., 2021). Moxifloxacin is the enabling component, providing the potent sterilizing activity necessary to achieve non-inferiority while shortening the treatment duration by one-third (Dorman et al., 2021; Gillespie, 2016). For patients aged 14 years or older with RR/MDR-TB, treatment has been dramatically shortened from previous 15–18 months courses to just six months through all-oral regimens (Jing et al., 2025). The preferred strategy for patients who remain fluoroquinolone-susceptible is the six-month BPaLM regimen (Bedaquiline, Pretomanid, Linezolid, and Moxifloxacin). Conversely, for patients classified as Pre-XDR-TB, those confirmed to have FQ resistance or intolerance, moxifloxacin is systematically omitted to avoid toxicity without benefit, resulting in the BPaL regimen (Bedaquiline, Pretomanid, and Linezolid) (Kumar et al., 2025).

The accelerated use of FQ-containing regimens is supported by recent evidence from pivotal Phase 3 randomized controlled trials (RCTs). The landmark trial, published by (Dorman et al., 2021) in The New England Journal of Medicine, conclusively demonstrated that the four-month rifapentine-based regimen containing moxifloxacin was non-inferior to the standard six-month regimen for DS-TB, providing the necessary clinical foundation for the updated global guidance favoring regimen shortening.

Beyond *M. tuberculosis*, fluoroquinolones are also critical agents in treating infections caused by Nontuberculous Mycobacteria (NTM) (Nguyen et al., 2024). FQs, such as levofloxacin or moxifloxacin, serve as essential second-line substitutes in multi-drug NTM regimens. For instance, in *Mycobacterium kansasii* infections, they are recommended alternatives when patients exhibit rifampicin resistance or intolerance to first-line agents (Srivastava et al., 2022). Furthermore, FQs are integrated into multidrug regimens for *M. xenopi* pulmonary disease, where clinical and *in vitro* data show they demonstrate efficacy comparable to macrolides (Andrejak et al., 2024). The selection of FQs for NTM therapy is based on their intrinsic activity and acceptable safety profile, often optimized by pharmacokinetic-pharmacodynamic (PK/PD) principles to maximize drug effectiveness (Kumar et al., 2024). *Mycobacterium abscessus* is an escalating global concern, with isolation rates rising notably in bronchiectasis and cystic fibrosis populations, where prevalence reaches approximately 7.9% (Prieto et al., 2023). Clinical management is hindered by *M. abscessus* intrinsic resistance to standard drugs, particularly inducible macrolide resistance mediated by the erm(41) gene (Nash et al., 2009). Consequently, fluoroquinolones like sitafloxacin are indispensable; they target DNA gyrase with high potency and maintain efficacy against resistant isolates (He et al., 2021). Standard regimens involve a multidrug approach, incorporating amikacin, imipenem, and oral agents such as clofazimine and fluoroquinolones, to achieve sustained culture conversion (Takano et al., 2021; Xu et al., 2025).

Given the widespread integration of fluoroquinolones across the spectrum of mycobacterial disease, from enabling the shortest DS-TB regimen to establishing the therapeutic core for DR-TB (BPaLM), this drug class has emerged as the pharmacological linchpin of modern mycobacterial treatment. The pervasive selective pressure this creates for resistance, which immediately escalates a TB patient to a Pre-XDR diagnosis, makes the rigorous study of FQ mechanisms and stewardship paramount for preserving the longevity of this cornerstone therapy. Thus, a deeper understanding of the factors governing intrinsic susceptibility and the diverse mechanisms of resistance across various mycobacterial species is urgently needed to maximize the utility and longevity of fluoroquinolones in the face of escalating global mycobacterial drug resistance.

## 2 Materials and methods

### 2.1 MIC determination

The minimum inhibitory concentration values were determined using 7H9/OADC medium (Middlebrook, Difco, MD, USA) supplemented with 0.05% Tween 80 (*Mtb*) or Tyloxapol (*Mab*) in which *Mycobacterium tuberculosis* H37Rv and *M. abscessus* ATCC 19977 strains were cultured. Most of the tested fluoroquinolones were dissolved in dimethyl sulfoxide (DMSO). However, ciprofloxacin and moxifloxacin were dissolved in MiliQ water and then filtered. Studied compounds were added to the growth medium, and dilutions were performed. The final concentration of DMSO in the medium never passed 5% (vol/vol) and did not have an influence upon *Mtb* and *Mab* growth. Cultures of both mycobacteria were prepared (the McFarland standard 1.0 diluted 100 times) and added to the medium supplemented with compounds. The controls of medium and cultures were also included. MIC values were assessed using the Resazurin Microplate Assay (REMA) (Franzblau et al., 1998). The susceptibility of the tested cultures was verified based on visual observation of color change of resazurin – from blue to pink if the bacteria were metabolically active. The REMA test was conducted at least in three independent biological replicates using independent bacterial cultures.

### 2.2 *In vitro* cytotoxicity assay

The cytotoxicity assessment was performed in compliance with international standards (ISO 10993-5:2009(E)). During this procedure, L-929 mouse fibroblasts were applied, and cytotoxicity was assessed using the MTT assay. IC50 values were determined based on three independent replicates using concentration ranges tailored to each compound. Data were analyzed via non-linear regression (log(inhibitor) vs. normalized response) using GraphPad Prism version 11.0.0. Moreover, the cytotoxicity of the studied chemicals was also determined regarding human monocyte-derived macrophages (MDMs). However, these cells were incubated with the investigated fluoroquinolones for 48 hours.

### 2.3 BC assessment

The bactericidal concentrations were determined based on measurement of mycobacterial cultures optical density (OD) and estimation of colony forming units (CFU). Cultures of *Mtb* and *Mab* were diluted with rich medium (7H9/OADC/0.05% Tween 80 or Tyloxapol) to acquire OD_600_ = 0.1. Then, proper concentrations of the tested fluoroquinolones were added. The control without any compound was also included. Three OD measurement points were conducted: on day 1, after 7 and 14 days (*Mtb*) and on the first day of the experiment, after 3 and 7 days (*Mab*). To verify whether the FQ concentrations selected based on OD resulted in a 99% reduction in bacterial viability, mycobacterial cultures exposed to the selected concentrations of the compounds and the control were sampled at scheduled time points. The cultures were diluted in growth medium, plated on 7H10 medium supplemented with OADC and 0.5% glycerol, and incubated at 37 °C. Colony-forming units (CFU) were counted after three to five weeks for *Mtb* and approximately one week for *Mab*. To verify bactericidal activity of clinafloxacin, DHQ1, and sarafloxacin (DHQ2), a recombinant *Mtb* and *Mab* strains possessing stable luciferase expression (*M. tuberculosis* attB::pMV306LuxABCD and *M. abscessus* pMV306DIhsp+LuxG13) were utilized. The experiment was performed with rich medium (7H9/OADC/0.05% Tween 80) which was applied to dilute mycobacterial cultures to OD_600_ = 0.1. The determined concentrations of clinafloxacin, DHQ1, and DHQ2 were added and measurement of bioluminescence was performed for a week. Bioluminescence readings were realized with an Infinite^®^ M Plex multimode microplate reader (Tecan). Control without any compound was also included. Bactericidal concentrations assessment was conducted in three independent replicates. Sourced data were analyzed with Graph Pad Prism 10.5.0.

### 2.4 Antimycobacterial activity against mycobacterial biofilms

Biofilm formation by *Mtb and Mab* was performed as previously described (Kassem et al., 2024) including some modifications. *Mycobacterium tuberculosis and M. abscessus* strains were cultured to an OD_600_ of 1.0 in liquid rich medium (7H9/OADC/0.05% Tyloxapol). The cultures were diluted 1:100 (vol/vol) in Sauton’s medium supplemented with 0.1% zinc sulfate and added to 24-well plates at 2.5 mL (*Mtb*) or 2.0 mL (*Mab*) per well. To achieve biofilm development, plates were precisely sealed with two layers of parafilm. Incubation was conducted at 37 °C under humidified conditions for seven to nine weeks (*Mtb*) or about one to two weeks (*Mab*). After biofilm formation, the growth medium was exchanged with fresh medium enriched with 0.1% casitone (2.3 mL/well for *Mtb* and 2.0 mL/well for *Mab* biofilms) and various concentrations of the studied fluoroquinolones. Each plate contained control biofilms (approximately three to four wells) without the addition of the chemicals. Incubation was performed at 37 °C for 48 hours (plates sealed with parafilm). Activity of the tested compounds against mycobacterial biofilms was established via resazurin-based fluorescence measurement. To each well 375 µL (*Mtb*) or 300 µL (*Mab*) of 0.02% resazurin was added and incubated for 90 minutes (*Mtb*) or 30 minutes (*Mab*). Fluorescence readings were conducted using the Infinite^®^ M Plex multimode microplate reader (Tecan) (excitation: 560 nm, emission: 590 nm). Statistical analysis of data from three independent experiments was performed using one-way ANOVA (Tukey’s multiple comparisons test), and results were visualized with GraphPad Prism (version 11.0.0).

### 2.5 Preparation of human MDMs and estimation of the bactericidal effect of the tested fluoroquinolones on intracellularly growing mycobacteria

Activity of the tested fluoroquinolones against mycobacteria located inside human macrophages was determined according to procedure characterized by (Batran et al., 2024) with minor alterations. The human macrophages were acquired by the process of monocyte differentiation. Monocytes were isolated from buffy coats derived from healthy human blood donors (Regional Blood Donation Station, Łódź, Poland). To ensure that the investigated chemicals are safe for human macrophages, the MTT test was performed. The cell culture was exposed to different concentrations of compounds for 48 hours, and the level of cytotoxicity was determined. Subsequently, human macrophages were infected with *M. tuberculosis* and *M. abscessus* (pMV306DIhsp+LuxG13) at an MOI of 1:10. After 2 hours of incubation (phagocytosis), bacilli located outside macrophages were removed through gentamicin supplementation (1 g/L). The samples were rinsed using Iscove’s medium with 2% human AB serum (Sigma, St. Louis, MO, USA). Thereafter, human macrophages infected with mycobacteria were incubated for 48 hours (37 °C, 10% CO2-90% air) in growth medium with various fluoroquinolone concentrations (for *Mab*: DHQ3 and sitafloxacin at concentrations of 7.5 µg/mL and 15.0 µg/mL; for *Mtb*: moxifloxacin, DHQ1, DHQ2 and DHQ3 at concentrations of 0.3 µg/mL, 0.5 µg/mL, 3.0 µg/mL and 0.35 µg/mL, respectively). Untreated control samples were included in the assay. Lysis of macrophages was performed using 0.1% SDS. Mycobacterial ability to survive inside human macrophages was evaluated on the grounds of the CFU method (*M. tuberculosis*) or bioluminescence measurement (*M. abscessus* pMV306DIhsp+LuxG13). Statistical analysis of data from three independent experiments was performed using one-way ANOVA (Dunnett’s multiple comparisons test), and results were visualized with GraphPad Prism (version 11.0.0).

### 2.6 Identification of molecular target by obtaining *M. tuberculosis* and *M. abscessus* mutants resistant to the studied compounds

*Mycobacterium tuberculosis* and *M. abscessus* mutants were obtained using solid 7H10/OADC (Middlebrook, Difco, MD, USA) medium with 0.5% glycerol and properly higher concentrations of the tested fluoroquinolones. Concentrations were established based on bactericidal values. The liquid culture was centrifuged to acquire OD_600_ = 4.0 and then added onto prepared 7H10 medium with compound. After 5 weeks (*Mtb*) single colonies were passaged to 7H10 medium supplemented with tested fluoroquinolone. The procedure was repeated once again, and then selected mutants were passaged to liquid 7H9/OADC medium (Middlebrook, Difco, MD, USA) enriched with 0.05% Tween 80 and determined concentrations of the tested compounds. Due to difficulties with selecting proper *Mab* mutants resistant to DHQ3, the *in vitro* evolution test, previously described by (Martini et al., 2024), was conducted with some modifications adapted to *M. abscessus*. The resistance levels were evaluated by the REMA test mentioned above. Chromosomal DNA was isolated using DNAzol reagent, and Sanger sequencing and/or Whole Genome Sequencing were performed. Sourced data were analyzed using Geneious Prime (Sanger sequencing) or BreSeq (WGS) software as described previously (Korycka-Machala et al., 2019, 2020). The WGS data was deposited in GenBank under accession no. PRJNA1404621.

### 2.7 Homology model

The homology models of DNA gyrase subunits A and B from *M. abscessus* (UniProt IDs: B1ME58 and X8DHG2, respectively) were prepared using tools available through the Max Planck Institute Bioinformatics Toolkit (MPI Toolkit) (Gabler et al., 2020; Zimmermann et al., 2018). First, a sequence-to-structure homology search was performed using HHpred (Hildebrand et al., 2009). The crystal structure of the DNA gyrase ternary complex from *M. tuberculosis H37Rv* (PDB ID: 5BTD) (Blower et al., 2016) was selected as the modeling template. A structural model was then generated using MODELLER (Webb and Sali, 2016). Subsequently, the *M. abscessus* ternary complex, comprising two GyrA and two GyrB subunits, was manually assembled following the architecture of the 5BTD complex. The coordinates of the two DNA strands, two magnesium ions, and associated water molecules were adopted from the crystal structure to preserve the hydrogen-bond network supporting the canonical water–Mg²^+^ bridge. The model was prepared in two variants: (i) Tyr129 in the unmodified state (TYR129) and (ii) Tyr129 modeled as a covalent phosphotyrosine linkage to DNA (PTR129).

### 2.8 Molecular docking analysis and theoretical binding strength evaluation

The two DNA gyrase structures were used as receptors for molecular docking: (a) the crystal structure from *M. tuberculosis* H37Rv (PDB ID: 5BTD) and (b) a homology model of *M. abscessus*. DNA, water molecules, and two Mg²^+^ ions were retained in both receptor structures during docking. Each receptor was prepared in two variants in Maestro (Schrödinger Release 2024-2, Schrödinger, LLC, New York, NY, 2024), with Tyr129 either unmodified (TYR129) or modeled as a covalent phosphotyrosine adduct linked to DNA (PTR129). Protein structures were prepared using the Schrödinger Protein Preparation Workflow (Sastry et al., 2013). Protonation states were assigned at pH 7.4 with hydrogen-bond optimization enabled (PROPKA), followed by an all-atom restrained energy minimization using the OPLS4 force field with a maximum RMSD of 0.3 Å (Lu et al., 2021). Three-dimensional structures of fluoroquinolones were downloaded from PubChem (Kim et al., 2025) and prepared using LigPrep (Schrödinger Release 2024-2). Ligand preparation used the OPLS4 force field, and ionization states were generated at pH 7.0 ± 2.0 using Epik Classic. Molecular docking was performed in Glide extra-precision (XP) mode (Friesner et al., 2006). Molecular graphics and visualizations were generated using PyMOL (version 3.1.0; Schrödinger, LLC).

## 3 Results

### 3.1 Screening and *in vitro* characterization of active fluoroquinolone compounds presenting antimycobacterial activity

A comprehensive phenotypic screening campaign was initiated to identify compounds with novel antimycobacterial activity. The screening was conducted using the OTAVA (Otava Chemicals Ltd.) 579 compounds that were preselected by using machine learning based on the similarity to the known anti-TB drugs and ECBD pilot-library containing 5,015 compounds including 2,464 bioactive compounds (European Chemical Biology Database, ECBD Pilot Compounds) against a reference strain of *M. tuberculosis* H37Rv and the rapidly growing non-tuberculous mycobacterium, *M. abscessus* ATCC 19977. The initial screen utilized a fixed compound concentration of 125 μg/mL and 100 μM for OTAVA and ECBD, respectively, in the REMA test to assess growth inhibition. This initial screen successfully identified a series of fluoroquinolone derivatives, including some with previously uncharacterized activity against *M. tuberculosis*. Subsequent analysis revealed a clear and significant difference in the susceptibility of the two mycobacterial species to the identified compounds. All the selected fluoroquinolones demonstrated highly potent activity against *M. tuberculosis*, with Minimum Inhibitory Concentration (MIC) values equal to or lower than 1 μg/mL. In stark contrast, none of the same compounds, with the notable exception of sitafloxacin, which served as a reference drug (He et al., 2021; Takano et al., 2021) inhibited *M. abscessus* growth at these concentrations. Five of the active compounds, sitafloxacin, clinafloxacin, gatifloxacin, ciprofloxacin, and moxifloxacin exhibited MIC values below 10 μg/mL against *M. abscessus* (**Table 1**). This disparity in susceptibility between the two strains represents a central and important observation from the screening results. Sitafloxacin emerged as the most potent compound against *M. tuberculosis* and *M. abscessus*, with a MIC of 0.0156 μg/mL and 0.33 μg/mL, respectively. The profound difference in susceptibility suggests that while these compounds likely target a conserved mechanism (fluoroquinolones are known to inhibit DNA gyrase), additional factors must contribute to the observed variation in efficacy between the two species (Takiff et al., 1994).

**Table 1:** *In vitro* susceptibility of *M. tuberculosis* and *M. abscessus* to selected fluoroquinolones.

Compound	*M. tuberculosis* MIC (μg/mL)	*M. tuberculosis* BC (μg/mL)	*M. abscessus* MIC (μg/mL)	*M. abscessus* BC (μg/mL)
DHQ1	0.1 ± 0.03 (n=5)	0.5	36.0 ± 8.94 (n=5)	100
Sarafloxacin (DHQ2)	1.4 ± 0.55 (n=5)	3.0	80.0 ± 0 (n=4)	>100
Gatifloxacin (DHQ3)	0.0625 ± 0 (n=4)	0.35	1.72 ± 0.91 (n=22)	7.5
Gemifloxacin	0.175 ± 0 (n=3)	ND	>39.0 (n=3)	ND
Clinafloxacin	0.084 ± 0 (n=3)	0.37	2.29 ± 0.99 (n=9)	12.8
Ciprofloxacin	0.44 ± 0.11 (n=3)	1.0	5.71 ± 2.14 (n=7)	12.0
Sitafloxacin	0.0156 ± 0 (n=3)	0.05	0.33 ± 0.12 (n=17)	1.5
Moxifloxacin	0.0625 ± 0 (n=3)	0.3	2.63 ± 1.19 (n=8)	16.0

ND, not determined.

Following the initial screening, a more detailed characterization of the lead compounds was performed by determining their bactericidal concentration (BC) values. The BC value was defined as the lowest concentration leading to a 99% reduction in viable bacteria, as quantified by Colony Forming Unit (CFU) analysis or by bioluminescence measurement (for clinafloxacin, DHQ1, and DHQ2) (**Table 1**; **Supplementary Figures 1**–**3**).

The results confirm the initial findings from the screening campaign, with all tested compounds showing significantly higher MIC and BC values against *M. abscessus* compared to *M. tuberculosis*. Sitafloxacin was the most potent agent overall, with a MIC of 0.0156 μg/mL and a BC of 0.05 μg/mL against *M. tuberculosis*, and a MIC of 0.33 μg/mL and a BC of 1.5 μg/mL against *M. abscessus*. A closer inspection of the data reveals the relationship between inhibitory and bactericidal concentrations. For instance, DHQ1’s BC for *M. tuberculosis* (0.5 μg/mL) is five times its MIC (0.1 μg/mL), indicating a bactericidal effect at a relatively low concentration, which is a desirable characteristic for an antimicrobial agent. Similarly, DHQ3 (gatifloxacin) has a BC of 0.35 μg/mL against *M. tuberculosis* and 7.5 μg/mL against *M. abscessus*, which are approximately 5.6-fold and 5-fold higher than their respective MICs, respectively. These relationships underscore the potent bactericidal nature of these compounds at concentrations close to their inhibitory values.

Further, the selected compounds were assessed for their cytotoxicity using the MTT assay on L929 mouse fibroblasts, a standard method for evaluating cytotoxicity in accordance with international standards (ISO 10993-5:2009(E), designed to test the biocompatibility of inert medical devices, materials, and their extracts. The results of the cytotoxicity evaluation were highly favorable. The fifty-percent inhibitory concentration (IC50​) values for all tested compounds DHQ1, sarafloxacin, and gatifloxacin were found to be 274–7248 and 5–155 times higher for *M. tuberculosis* and *M. abscessus*, respectively, than their respective MIC values (**Supplementary Table 1**). Additionally, the compounds were evaluated for toxicity against human monocyte-derived macrophages (hMDMs) during intracellular killing experiments. In these assays, the new compounds selected from the library were tested at concentrations of 1x, 2x, and 4x the Minimum Bactericidal Concentration (MBC). At the highest concentration (4x MBC), gatifloxacin showed 27% cytotoxicity and sarafloxacin showed 11%, while DHQ1 exhibited no toxicity. The remaining compounds were tested at the concentrations used in the intracellular killing assay and also exhibited no toxicity (**Supplementary Table 2**).

### 3.2 Activity against physiologically relevant mycobacterial states

**Figure 1:**
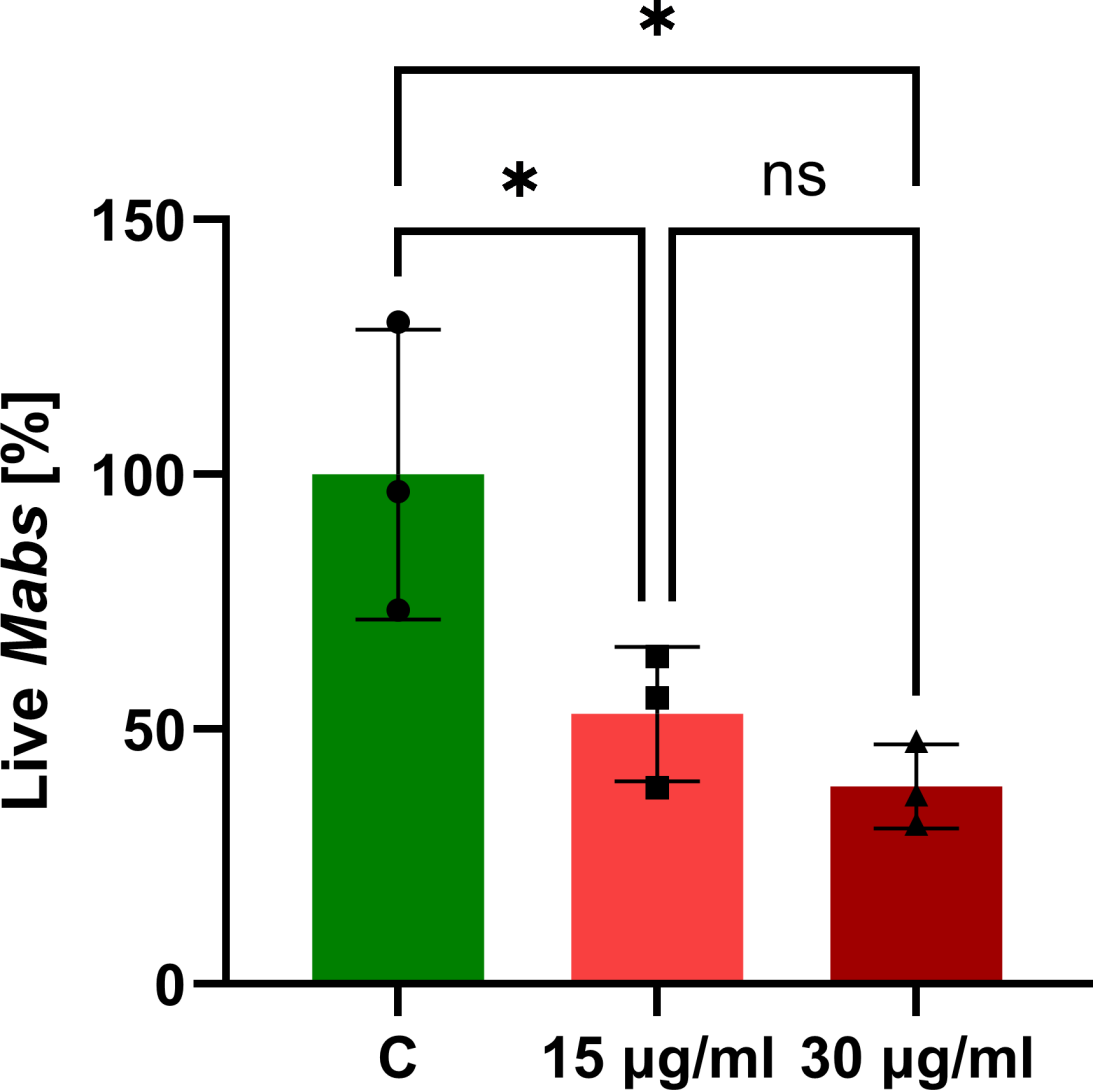
Viability of mature *M. abscessus* biofilm after exposure to various concentrations of gatifloxacin (15 μg/mL and 30 μg/mL). Statistical analysis was performed using the Ordinary one-way ANOVA powerful indicator of their’s multiple comparisons test. Statistical significance is presented as * (0.0492 – 15 μg/mL) and * (0.0165 – 30 μg/mL). NS, no statistically significant.

Mycobacteria, including *M. tuberculosis* and *M. abscessus*, are known to form complex multicellular structures called biofilms, particularly in chronic infections (Qvist et al., 2015). These biofilms provide a protective matrix that makes the bacteria significantly more resistant to antimicrobial agents and host immune responses. Consequently, evaluating a compound’s activity against biofilm-forming mycobacteria is crucial for predicting its potential clinical efficacy. Some of the tested compounds that showed antimycobacterial activity were therefore evaluated for their ability to eliminate mycobacteria in a biofilm state. The results demonstrated that all tested compounds exhibited bactericidal activity against biofilms, though at higher concentrations than those required to kill planktonic cells. Specifically, bactericidal activity was achieved at concentrations approximately 2 times the standard BC values. For *M. abscessus*, gatifloxacin was shown to significantly reduce the percentage of live bacteria within the biofilm at concentrations of 15 μg/mL and 30 μg/mL compared to the untreated control. While both concentrations were effective, the difference in live bacteria between the 15 μg/mL and 30 μg/mL treatments was not statistically significant, suggesting that efficacy plateaus at a certain concentration (**Figure 1**).

**Figure 2:**
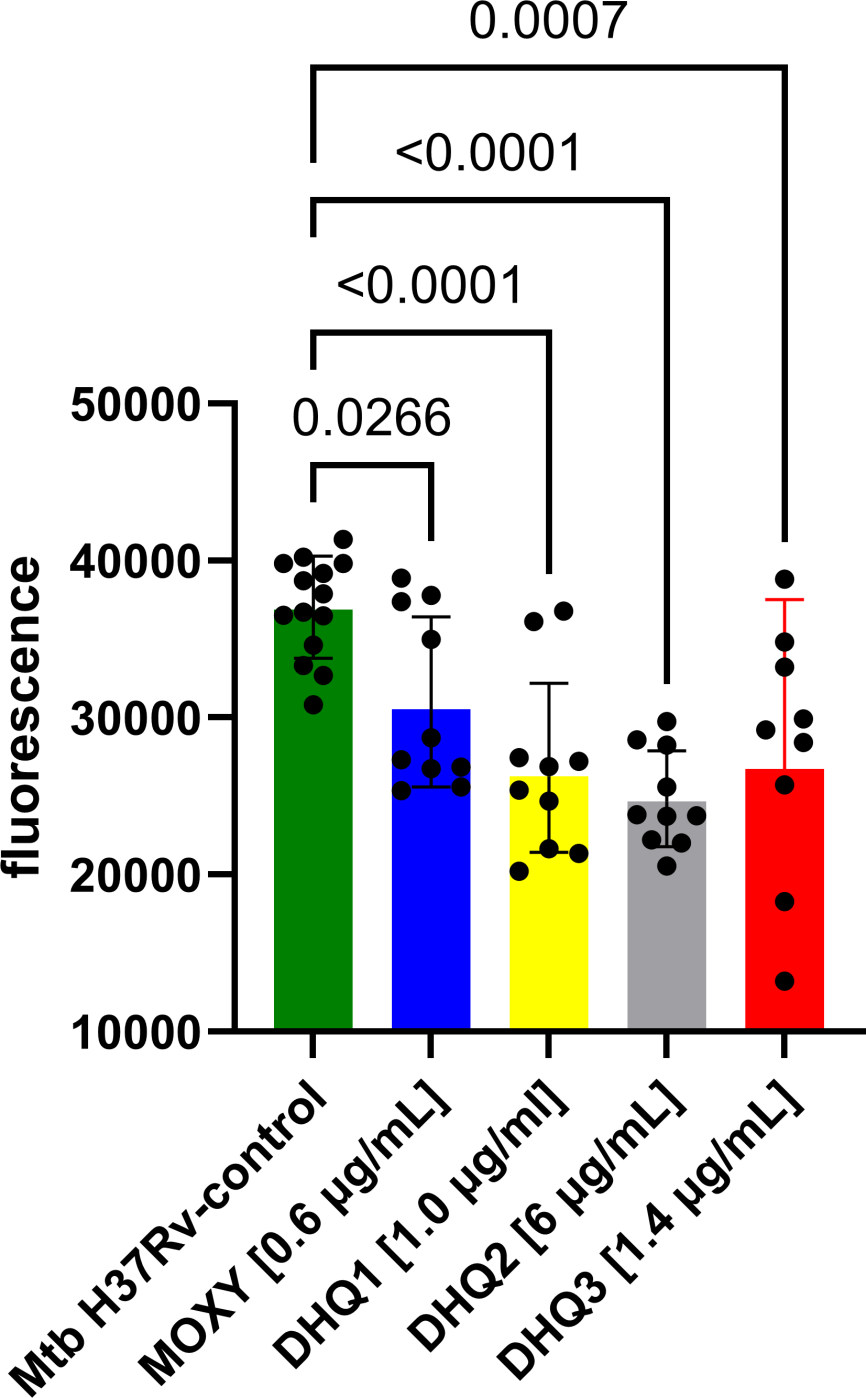
Determination of antitubercular activity of the tested fluoroquinolones against mature *Mtb* biofilm. Statistical analysis was conducted using the Ordinary one-way ANOVA Dunnett’s multiple comparisons test. Statistical significance (P<0.05) is displayed on graphs.

Against *M. tuberculosis* biofilms, DHQ1, sarafloxacin (DHQ2), gatifloxacin (DHQ3), and moxifloxacin all caused a statistically significant reduction in bacterial viability, as measured by fluorescence, at specific concentrations. For example, moxifloxacin at 0.6 μg/mL, DHQ1, at 1.0 μg/mL, DHQ2 at 6.0 μg/mL, DHQ3 at 1.4 μg/mL all showed a profound reduction in fluorescence, indicating a significant killing effect (**Figure 2**).

**Figure 3:**
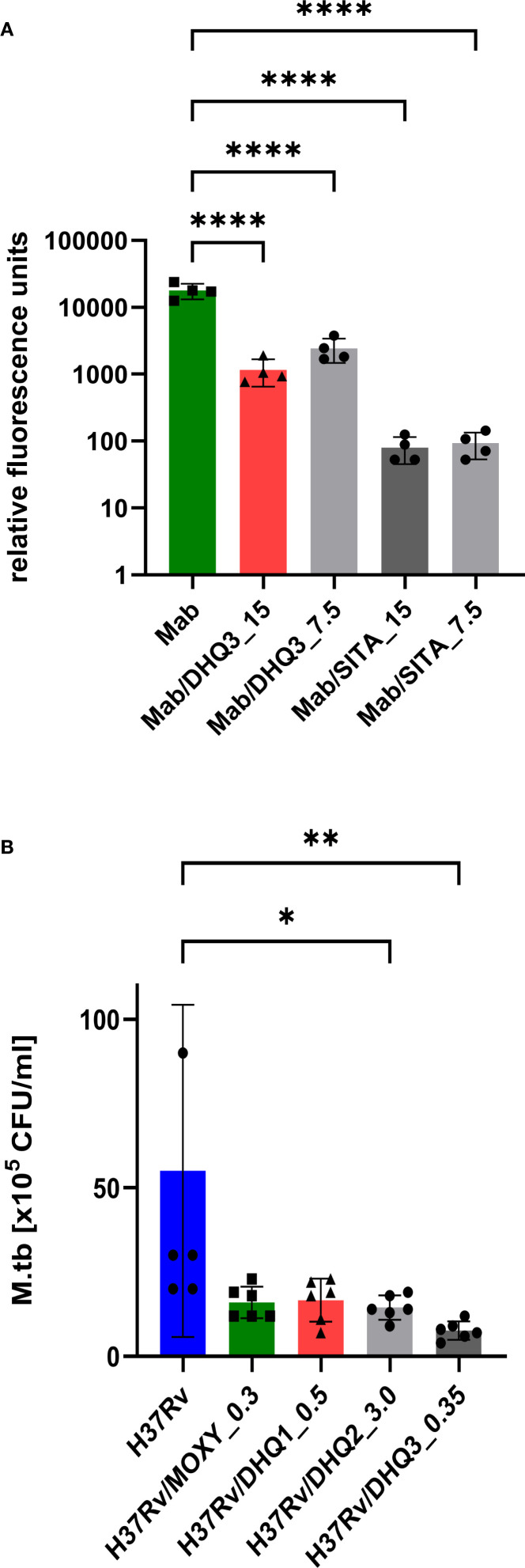
The viability of *M. abscessus* **(A)** and *M. tuberculosis* **(B)** deposited in human macrophages after treatment with selected fluoroquinolones. A – statistical analysis was executed by the Ordinary one-way ANOVA, Dunnett’s multiple comparisons test. Statistical significance is shown as **** (<0.0001 – 15 µg/mL DHQ3, <0.0001 – 7.5 µg/mL DHQ3, <0.0001 – 15 µg/mL sitafloxacin, <0.0001 – 7.5 µg/mL sitafloxacin). B – statistical analysis was performed by the Ordinary one-way ANOVA, Dunnett’s multiple comparisons test. Statistical significance is presented as * (0.0201 – Moxy; 0.0226 – DHQ1; 0.0153 - DHQ2) and ** (0.0043 - DHQ3).

Another critical consideration for developing effective treatments for mycobacterial infections is the fact that both *M. tuberculosis* and *M. abscessus* are facultative intracellular pathogens. They can survive and replicate within host macrophages, which are normally responsible for their clearance (Radhakrishnan and Sundaramurthy, 2026). Therefore, an effective drug must not only be potent but also capable of penetrating host cells and retaining its activity within the hostile, acidic, and nutrient-poor environment of the phagolysosome. To address this, the antimycobacterial activity of the selected fluoroquinolones was evaluated against mycobacteria located within human monocyte-derived macrophages (MDM). The evaluation was performed using two different methodologies tailored to the specific growth characteristics of each species. The viability of intracellular *M. abscessus* was assessed using a recombined strain with stable luciferase gene expression, which allows for the quantification of viable bacteria through relative fluorescence units. In contrast, the viability of intracellular *M. tuberculosis* was enumerated by a CFU analysis. The results from both assays were highly encouraging. All tested drugs effectively reduced the burden of both *M. abscessus* and *M. tuberculosis* present within the phagocytic cells in a statistically significant manner. Specifically, gatifloxacin and sitafloxacin caused a significant reduction in the relative fluorescence units of intracellular *M. abscessus* at both 7.5 μg/mL and 15 μg/mL concentrations compared to the control (**Figure 3A**). Similarly, treatment with DHQ1, sarafloxacin (DHQ2), gatifloxacin (DHQ3), and moxifloxacin in bactericidal concentrations (0.5; 3.0; 0.35, and 0.3, respectively) led to a significant reduction in the number of viable intracellular *M. tuberculosis* cells (**Figure 3B**). While the results validate that these compounds retain their activity within macrophages for each bacterium separately, the variation in viability readouts and drug panels, namely fluorescence units versus colony counts, precludes a direct cross-species comparison of their efficacy or mechanistic behavior concerning phagolysosomal efflux. Nevertheless, the ability of these compounds to penetrate host cells and retain bactericidal efficacy within the intracellular environment suggests their potential utility in a cellular context. It confirms that all tested compounds cross the macrophage cell membrane and demonstrate efficacy against intracellularly located bacilli.

### 3.3 Mode of action and resistance analysis

Fluoroquinolones primarily exert their antimicrobial effect by targeting DNA gyrase, a type II topoisomerase crucial for DNA replication, transcription and repair in mycobacteria (Manjunatha et al., 2002). The mechanism by which mycobacteria develop resistance to these agents is generally associated with the accumulation of specific mutations within the genes encoding the gyrase subunits, *gyrA* and *gyrB* (Takiff et al., 1994). To confirm the role of DNA gyrase as the molecular target for the novel compounds, we assessed the MIC values of the tested fluoroquinolones against recombinant *M. tuberculosis* strains carrying key resistance-conferring mutations in the *gyrA* gene: A90V and D94G. We also tested the compounds against a *M. abscessus* mutant strain containing the D96G *gyrA* mutation. The results demonstrated that the presence of these known resistance mutations led to a significant increase in the MIC values for all tested compounds when compared to their activity against the wild-type strains (**Table 2**).

**Table 2:** MIC values of the tested compounds for *M. tuberculosis* and *M. abscessus* mutants carrying mutations in the *gyrA* gene.

Compound	MIC [µg/mL]		
	*M. abscessus* GyrA (D96G)	*M. tuberculosis* GyrA (A90V)	*M. tuberculosis* GyrA (D94G)
DHQ1	>160.0	0.5	1.0
Sarafloxacin (DHQ2)	>160.0	8.0	32.0
Gatifloxacin (DHQ3)	40.0	0.5	1.0
Moxifloxacin	128.0	0.5	1.0
Clinafloxacin	18.3	0.67	0.67
Ciprofloxacin	>256.0	2.0	4.0
Sitafloxacin	1.875	0.0625	0.125

average data of n=3.

For example, the activity of DHQ2 (sarafloxacin) dropped considerably, with MICs rising from 1.4 μg/mL (wild-type *M. tuberculosis*) to 8.0 μg/mL and 32.0 μg/mL against the A90V and D94G mutants, respectively. Similarly, DHQ3 (gatifloxacin) and moxifloxacin, highly potent against wild-type *M. tuberculosis* (0.0625 μg/mL for both), showed 8- to 16-fold increases in MIC against the A90V and D94G mutants. Against the *M. abscessus* D96G mutant, compounds DHQ1, DHQ2, moxifloxacin, and ciprofloxacin demonstrated vastly reduced potency, with MICs climbing to >160.0 μg/mL, 128.0 μg/mL, and >256.0 μg/mL, respectively. Notably, sitafloxacin retained the highest relative potency against all tested mutant strains. These findings confirm that the primary molecular target of the novel compounds is, indeed, DNA gyrase, and that point mutations in the gyrase A subunit substantially diminish their efficacy.

To further verify that gyrase mutations represent the dominant mechanism for acquired resistance, we selected resistant mutants of *M. tuberculosis* and *M. abscessus* by culturing them on media containing selected fluoroquinolones at high concentrations (≥10× the initial MIC). Subsequent Sanger sequencing and/or whole-genome sequencing (WGS) analysis of these selected resistant strains confirmed the presence of point mutations in the *gyrA* and/or *gyrB* genes in all obtained *M. tuberculosis* and *M. abscessus* mutants. Eight mutants selected on a medium with a concentration of 21 μg/mL of compound DHQ2, and four mutants selected in the presence of 10.5 μg/mL of compound DHQ2, underwent whole-genome sequencing. In all eight mutants selected on the medium with the higher concentration of the tested compound, the N499Y mutation in DNA gyrase subunit B was identified. In three mutants selected in the presence of the lower concentration of the compound, the N499K mutation was identified in the same gene, and in one case, D94G in the DNA gyrase subunit A gene. No other mutations that could be linked to drug resistance or compensatory mutations in topoisomerase genes were observed in any of the mutants.

Analysis of the *M. tuberculosis* mutants revealed several key substitutions (**Table 3**). Mutants selected in the presence of DHQ2 (sarafloxacin) and DHQ3 (gatifloxacin) were uniquely characterized by a substitution in the GyrB subunit at position 499 (N499K or N499Y), in addition to GyrA mutations such as D94G, D94E, or D94F. The remaining *M. tuberculosis* mutants, selected with DHQ1, moxifloxacin, and ciprofloxacin, primarily accumulated substitutions at the common positions 90 and 94 of the GyrA subunit (e.g., A90V, D94N, D94G). The *M. abscessus* mutants selected in the presence of DHQ3 (gatifloxacin) also acquired substitutions in the *gyrA* gene, specifically G90C and D96G. Crucially, the analysis concluded that no fundamental differences were observed in the molecular mechanisms by which *M. tuberculosis* and *M. abscessus* acquire fluoroquinolone resistance, suggesting a shared structural basis for this acquired resistance.

**Table 3:** MIC values and the presence of mutations in selected *M. tuberculosis* and *M. abscessus* resistant strains.

*Mtb* mutants (applied concentration)	MIC [µg/mL]	*gyrA* mutation(s)	*gyrB* mutation(s)
DHQ1 (1.5; **2.5**; **5.0**)	10.0; 20.0	A90V, D94G	–
Sarafloxacin (DHQ2) (**10.5**; **21.0**; **30.0**)	84.0; 60.0	D94G, D94E, D94F	N499K, N499Y
Gatifloxacin (DHQ3) (**0.5**)	1.0	–	N499K
Moxifloxacin (**0.6**)	1.2	D94N	–
Ciprofloxacin (**2.5**)	10.0	D94N	–
*M. abscessus* mutants (applied concentrations)	MIC [µg/mL]	*gyrA* mutation(s)	*gyrB* mutation(s)
Gatifloxacin (DHQ3) (**45.0**)	32.0-256.0	G90C, D96G	–

### 3.4 Molecular docking and theoretical binding strength

To investigate the basis of the differential susceptibility - particularly the substantially higher MIC values observed for *M. abscessus* - we performed molecular docking of the tested compounds to GyrA/GyrB–DNA complexes from *M. tuberculosis* and *M. abscessus*. DNA gyrase subunits are highly conserved between these species, sharing 89.2% and 91.3% sequence identity for GyrA and GyrB, respectively, and 95.1% and 93.7% sequence similarity. This level of conservation would be expected to support broadly comparable inhibitor interactions, particularly because the fluoroquinolone (FQ) binding sites are fully conserved between the two species (**Supplementary Figure 4**). We therefore used docking to assess whether subtle primary-sequence differences outside the conserved binding pocket could drive conformational differences that alter interactions with FQ derivatives.

Docking simulations indicated that all tested FQs adopt broadly similar binding modes in the *M. tuberculosis* and *M. abscessus* gyrase–DNA complexes. In the docked poses, the 4-oxo-3-carboxyquinolone core intercalated at the DNA cleavage site and engaged in π–π stacking interactions with DNA bases, consistent with known FQs binding modes. In addition, the C3/C4 keto/carboxyl motif supported the canonical water–metal-ion (Mg²^+^) bridge interaction observed in co-crystal structures of FQs bound to bacterial type II topoisomerases, including *M. tuberculosis* (PDB: 5BTD) (**Supplementary Figures 5**, **6**) (Blower et al., 2016; Collins and Osheroff, 2024).

Despite these overall similarities in binding mode, comparison of docking scores suggested systematically weaker predicted binding for all compounds to the *M. abscessus* gyrase complex relative to the *M. tuberculosis* complex. Among the compounds evaluated *in silico*, DHQ1 exhibited the weakest predicted binding in both species (independent of the Tyr129 state). For *M. tuberculosis*, the highest-scoring ligands included ciprofloxacin, moxifloxacin, sitafloxacin, and the best scored gatifloxacin (-14.888 kcal/mol, PTR129). In contrast, the top-scoring ligands for *M. abscessus* included moxifloxacin (-11.165 kcal/mol, PTR129) and sarafloxacin (-10.984 kcal/mol, PTR129), with larger gaps relative to the best-scoring clinafloxacin (-12.018 kcal/mol, PTR129). Overall, the predicted differences in binding energy between species ranged from ~1.7 to ~5.1 kcal/mol for PTR129 and from ~0.2 to ~3.7 kcal/mol for TYR129, favoring binding to *M. tuberculosis* (**Table 4**; **Supplementary Table 3**). Although these predicted differences are broadly consistent with the experimental susceptibility trends, docking scores should be interpreted cautiously and are not expected to map quantitatively to MIC values (see Discussion).

**Table 4:** Theoretical binding strength of fluoroquinolones to the gyrase complexes (in kcal/mol) for covalent phosphotyrosine adduct linked to DNA (PTR129).

Compounds	*M. tuberculosis*	*M. abscessus*
DHQ1	-11.255	-6.604
Sarafloxacin (DHQ2)	-13.759	-10.965
Gatifloxacin (DHQ3)	-14.888	-10.912
Ciprofloxacin	-14.198	-10.412
Moxifloxacin	-14.567	-11.165
Gemifloxacin	-12.972	-10.600
Clinafloxacin	-13.732	-12.018
Sitafloxacin	-14.644	-10.769

The results for free Tyr129 (TYR129) are presented in **Supplementary Table 3**.

## 4 Discussion

The global challenge of tuberculosis (TB) control is increasingly defined by the efficiency of treatment, driven by the strategic integration of the fluoroquinolone (FQ) class, particularly moxifloxacin and levofloxacin. The comprehensive *in vitro* and *ex vivo* characterization presented here confirms the potent antimycobacterial activity of FQ derivatives against *M. tuberculosis* and highlights their potential utility against the highly recalcitrant non-tuberculous mycobacterium, *M. abscessus*, while simultaneously elucidating the profound differences in drug susceptibility between the two species (Orgeur et al., 2024; Nguyen et al., 2024).

Our findings, which demonstrate that FQ derivatives exhibit strong bactericidal activity against *M. tuberculosis* (MIC values as low as 0.0156 µg/mL for sitafloxacin), reinforce the clinical evidence that has established FQs as the pharmacological linchpin of modern TB management. Clinically, moxifloxacin is the enabling component of the four-month rifapentine-based regimen for drug-susceptible TB, shortening the standard treatment course by one-third (Dorman et al., 2021; Gillespie, 2016). Moreover, FQ susceptibility is paramount for drug-resistant TB, as FQ resistance immediately escalates a patient’s diagnosis to Pre-Extensively Drug-Resistant TB (Pre-XDR-TB) (Alagna et al., 2021). For FQ-susceptible patients, moxifloxacin is mandated within the six-month, all-oral BPaLM regimen, signifying its indispensable sterilizing activity in accelerating therapy (Jing et al., 2025). The robust activity demonstrated in our assays, specifically the significant killing of *M. tuberculosis* within human macrophages and in protective biofilm structures, aligns with the pharmacological rationale for using FQs in TB therapy. Since *M. tuberculosis* is a facultative intracellular pathogen and often persists in biofilms during chronic infection, the compounds’ ability to penetrate host cells and retain bactericidal efficacy at tested concentrations suggests potential effectiveness that requires further *in vivo* validation.

In contrast to *M. tuberculosis*, *M. abscessus* is generally recognized as intrinsically resistant to most anti-TB drugs, including FQs (Daley et al., 2020). While FQs are sometimes used as essential second-line agents for rifampicin-resistant *M. kansasii* and have shown efficacy comparable to macrolides in *M. xenopi* regimens (Daley et al., 2020), the role of FQs in primary *M. abscessus* treatment is limited.

The phenotypic data presented here underscores this disparity: all tested FQ compounds exhibited markedly higher MIC and bactericidal concentration (BC) values against *M. abscessus* compared to *M. tuberculosis*. For instance, while moxifloxacin was highly potent against *M. tuberculosis* (MIC 0.0625 µg/mL), its potency was significantly diminished against *M. abscessus* (MIC 2.63 µg/mL). Only sitafloxacin and gatifloxacin demonstrated sufficient potency to warrant further consideration against *Mab*, particularly their ability to significantly reduce intracellular and biofilm viability at concentrations that justify further investigation in animal models.

The profound difference in intrinsic susceptibility between *M. tuberculosis* and *M. abscessus* represents a key finding and necessitates a discussion of the underlying mechanisms. Fluoroquinolones primarily exert their effect by targeting DNA gyrase, inhibiting replication, and repair (Hooper, 2001). Our analysis confirmed that acquired resistance, whether in *M. tuberculosis* or *M. abscessus*, is overwhelmingly mediated by point mutations in the Quinolone Resistance Determining Regions (QRDRs) of the *gyrA* and *gyrB* subunits. Crucially, we observed no fundamental differences in the molecular mechanisms by which the two species acquire FQ resistance, with key substitutions identified in both *gyrA* (e.g., D94G) and *gyrB* (e.g., N499K/Y) for both pathogens.

Therefore, the vast disparity in intrinsic susceptibility must stem from factors other than the shared mechanism of acquired resistance. Molecular docking simulations indicated that fluoroquinolones (FQs) have slightly weaker predicted binding to the *M. abscessus* gyrase complex than to the *M. tuberculosis* gyrase. The predicted differences in binding energy ranged from ~1.7 to ~5.1 kcal/mol for Ptr129 and from ~0.2 to ~3.7 kcal/mol for Tyr129, which could translate into orders-of-magnitude differences in binding affinity. Although the *in silico* results were generally consistent with experimental observations, the remaining discrepancies may reflect several factors. Because the FQ-binding sites are 100% identical between the two species, differences in predicted binding may instead be driven by subtle differences in residue orientation. For example, Arg128 adopts two distinct conformations in the protein structures used for docking. In *M. tuberculosis*, Arg128 is oriented toward the C4 carboxyl group of the FQ scaffold, enabling a strong protein–ligand salt bridge and yielding more favorable predicted binding. In contrast, in *M. abscessus*, Arg128 is oriented such that it cannot interact with FQs. Determining whether this favorable/unfavorable Arg128 orientation reflects subtle differences in the primary sequence or is an artifact of homology modeling will require more exhaustive simulations (e.g., molecular dynamics) of the full GyrA/B–DNA–ligand complex. In addition, any direct correlation between predicted binding energies and experimental MIC values may be confounded by other biological factors (discussed below) and should be validated in future work using *in vitro* biophysical methods (e.g., to estimate K_D_ or IC_50_).

Furthermore, our *in silico* observations reveal distinct differences in the molecular docking of FQs to the *M. tuberculosis* and *M. abscessus* gyrases, aligning with published *in vitro* studies (Negatu et al., 2021; Ture et al., 2019). These studies demonstrate that the IC_50_ of moxifloxacin is 4-fold higher for *M. abscessus* gyrase (17 µM) compared to *M. tuberculosis* gyrase (4.2 µM), suggesting that this discrepancy stems from variations in drug-target binding affinities.

However, the observed differences in FQ MIC values between *M. tuberculosis* and *M. abscessus* may be driven by drug-target independent mechanisms. For example, factors that modulate the effective intracellular drug concentration, such as a cell envelope permeability barrier or active efflux pump systems, could contribute to the intrinsic resistance observed in *M. abscessus* (Hooper, 2001). However, verifying this would require experiments specifically aimed at a direct comparison of these factors in both bacterial species, which was beyond the scope of the studies described in this work. Further mechanistic studies should focus on structural modifications that bypass or inhibit the putative efflux mechanisms of *M. abscessus*, transforming high intrinsic activity into improved efficacy against resistant NTM pathogens.

## Data Availability

The datasets presented in this study can be found in GenBank under accession no. PRJNA1404621.
