# Supplementary material for: Fluoroquinolones as the linchpin of tuberculosis therapy: contrasting efficacy and resistance profiles in Mycobacterium tuberculosis versus Mycobacterium abscessus
Source: Front Cell Infect Microbiol. 2026 May 28;16:1833650. doi: 10.3389/fcimb.2026.1833650 (PMC13253442; doi:10.3389/fcimb.2026.1833650)
Supplement: Supplementary file 1 [file DataSheet1.pdf]

Fluoroquinolones as the Linchpin of Tuberculosis Therapy: Contrasting Efficacy and Resistance Profiles in *Mycobacterium tuberculosis* versus *M. abscessus*.

Magdalena Kuzioła[1, 2], Małgorzata Korycka-Machała[1], Daria Zygała-Pytlos[1, 2], Bożena Dziadek[3], Malwina Kawka[3], Norbert Odolczyk[4,5], Piotr Zielenkiewicz[4,5], Marcin Słomka[6] and Jarosław Dziadek[1#]

Supplementary materials:

Legends:

1. **Figure S1**. Evaluation of the bactericidal activity of the tested compounds through CFU analysis.
2. **Figure S2.** Assessment of the bactericidal activity of clinafloxacin against *M. abscessus* and *M. tuberculosis* based on luminescence measurements.
3. **Figure S3.** Assessment of the bactericidal activity of DHQ1 and DHQ2 against *M. abscessus* based on luminescence measurements.
4. **Table S1.** IC50 values for the tested compounds.
5. **Table S2.** Assessment of hMDM cytotoxicity.
6. **Table S3.** Theoretical binding strength of fluoroquinolones to the gyrase complexes (in kcal/mol) for free Tyr129 (TYR129).
7. **Figure S4.** Sequence alignment between *M. tuberculosis* and *M. abscessus* gyrases.
8. **Figure S5.** The schematic binding modes of docked FQs to the *M. abscessus* homology model.
9. **Figure S6.** 3D binding site view of best docked compound – clinafloxacin to the *M. abscessus* homology model structure with PTR129.

Figure S1

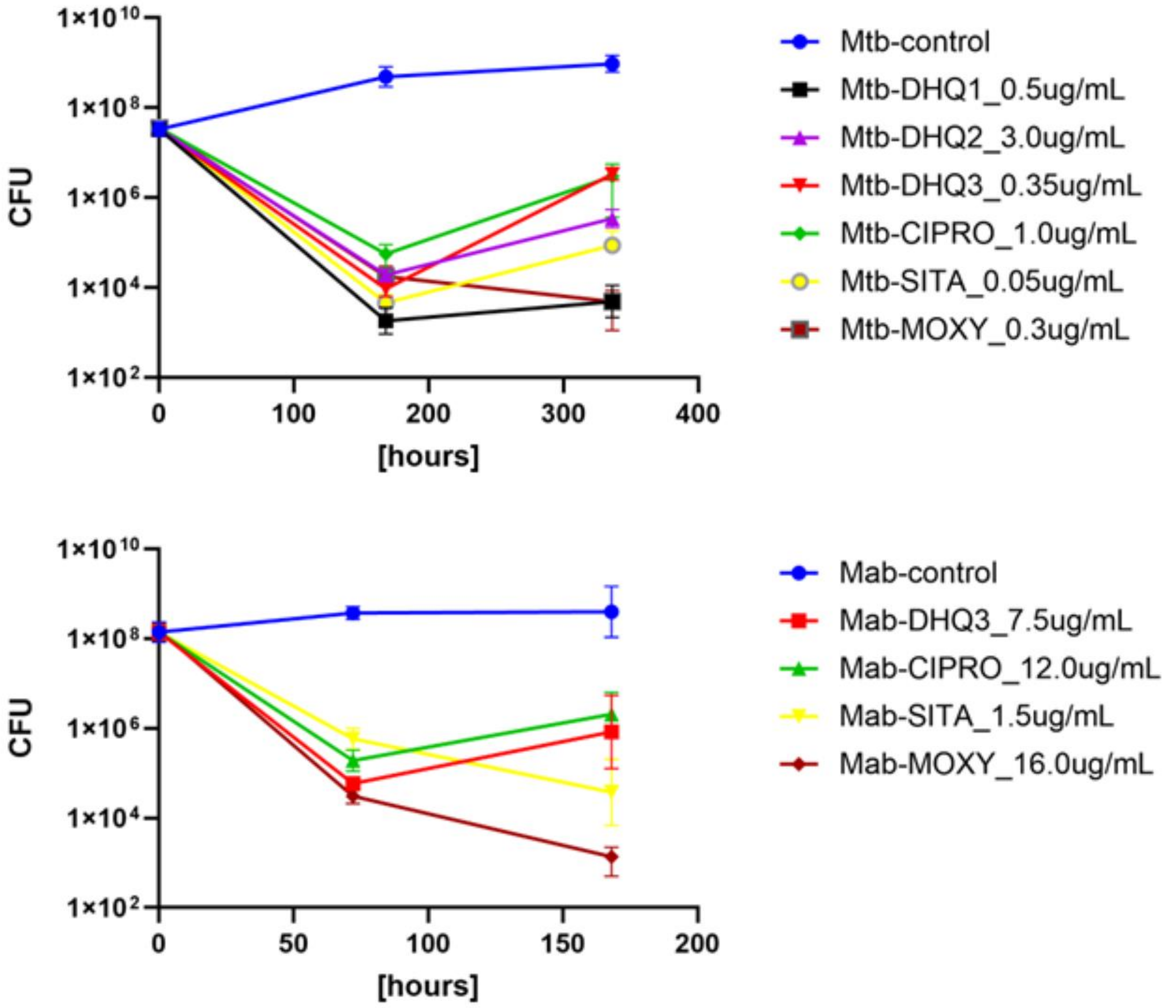

Figure S1. Evaluation of the bactericidal activity of the tested compounds through CFU analysis. The graph illustrates the bactericidal activity of the indicated compounds and the concentrations used against *M. tuberculosis* (upper panel) and *M. abscessus* (lower panel). The bactericidal concentration was defined as the concentration of a given compound that resulted in a 99% reduction in viable bacterial counts over a period of 336 hours (*M. tuberculosis*) or 168 hours (*M. abscessus*). Data visualization was performed based on three independent experiments using GraphPad Prism 9 software (version 10.5.0).

Figure S2

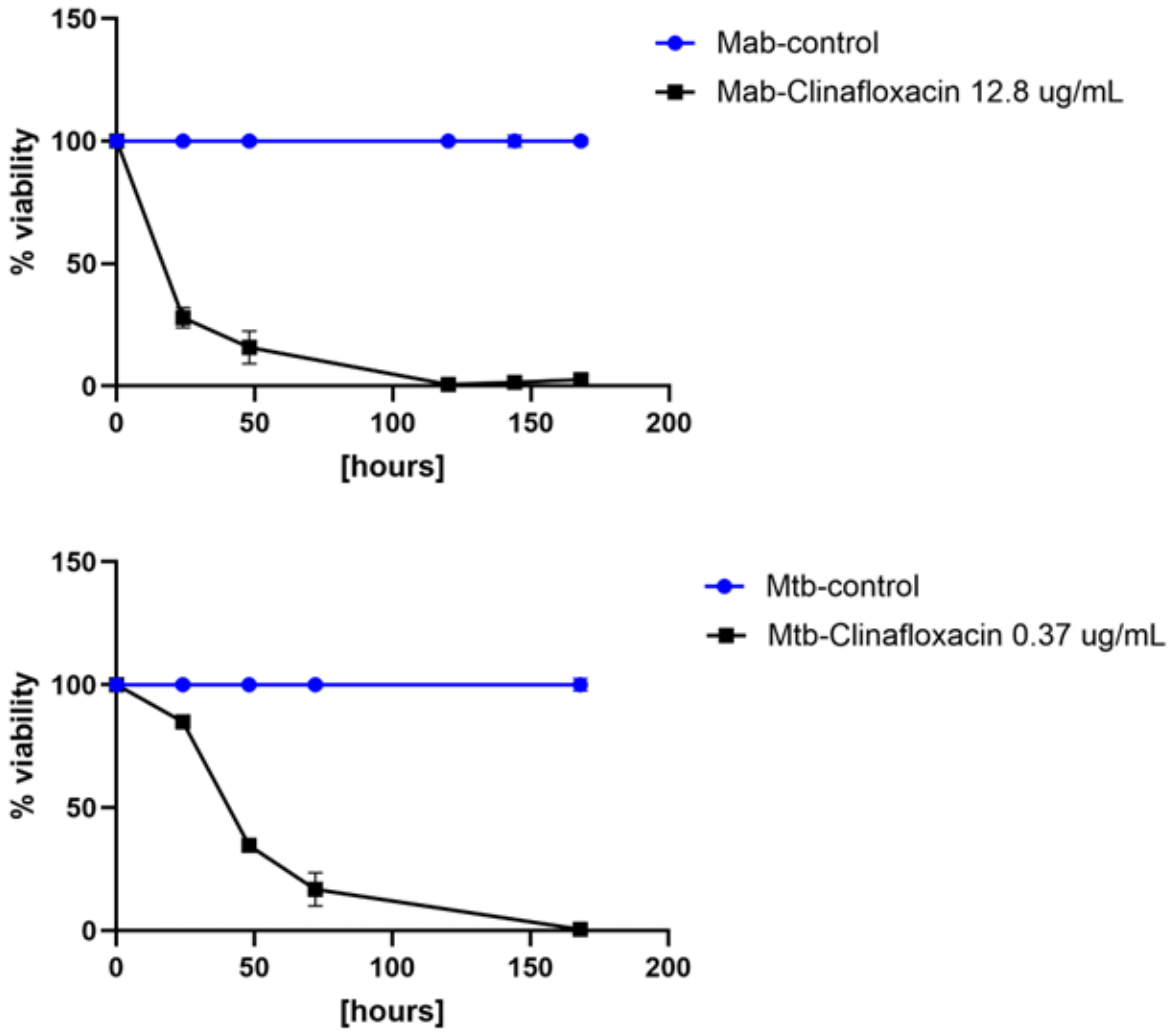

Figure S2. Assessment of the bactericidal activity of clinafloxacin against *M. abscessus* and *M. tuberculosis* based on luminescence measurements. The bactericidal concentration was defined as the level required to eliminate 99% of the bacterial population following 168 hours of incubation. Data visualization was performed based on 3 independent experiments using GraphPad Prism 9 software (version 10.5.0).

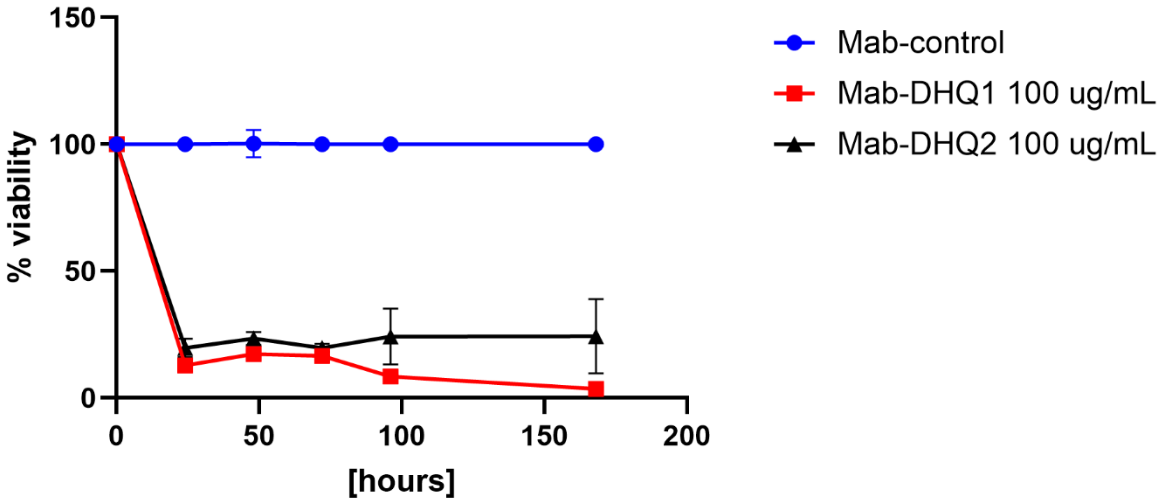

Figure S3. Assessment of the bactericidal activity of DHQ1 and DHQ2 against *M. abscessus* based on luminescence measurements. The bactericidal concentration was defined as the level required to eliminate 99% of the bacterial population following 168 hours of incubation. Data visualization was performed based on 3 independent experiments using GraphPad Prism 9 software (version 10.5.0).

Table S1. IC50 values for the tested compounds. MTT assay on L929 mouse fibroblasts with international standards (ISO 10993-5:2009(E) was applied.

**compound**	IC50	MIC-*Mtb*	MIC-*Mab*	IC50/MIC *Mtb*	IC50/MIC *Mab*
**DHQ1**	724.8	0.1	36.0	7248.0	20.13
**Sarafloxacin** (DHQ2)	383.4	1.4	80.0	273.86	4.79
**Gatifloxacin** (DHQ3)	266.6	0.0625	1.72	4265.6	155.0

Table S2. Assessment of hMDM cytotoxicity. Cytotoxicity was assessed via the MTT assay after hMDMs were incubated with the tested compounds for 48 hours.

**compound**	control	MBC	2xMBC	4xMBC
**DHQ1**	0%	0%	0%	0%
**Sarafloxacin** (DHQ2)	0%	9%	15%	11%
**Gatifloxacin** (DHQ3)	0%	14%	16%	26%

% of toxicity, MBC – minimal bactericidal concentration, control – no compound

Table S3. Theoretical binding strength of fluoroquinolones to the gyrase complexes (in kcal/mol) for free Tyr129 (TYR129).

	M. tuberculosis	***M. abscessus***
**DHQ1**	-11.499	-8.808
**Sarafloxacin** (DHQ2)	-12.786	-9.111
**Gatifloxacin** (DHQ3)	-13.482	-11.372
**Ciprofloxacin**	-13.091	-11.58
**Moxifloxacin**	-13.301	-10.855
**Gemifloxacin**	-12.311	-12.083
**Clinafloxacin**	-13.138	-11.898
**Sitafloxacin**	-11.787	-12.443

Figure S4. Sequence alignment between *M. tuberculosis* and *M. abscessus* gyrases. The residues of FQs' binding site are marked as red dots and TYR129/PTR129 is marked as green, top arrow. The sequence numbering is adopted from *M. tuberculosis* crystal structure – homology template modeling (PDB id: 5BTD). Prepared by ESPript v. 3.2 (Gouet et al., 2003).

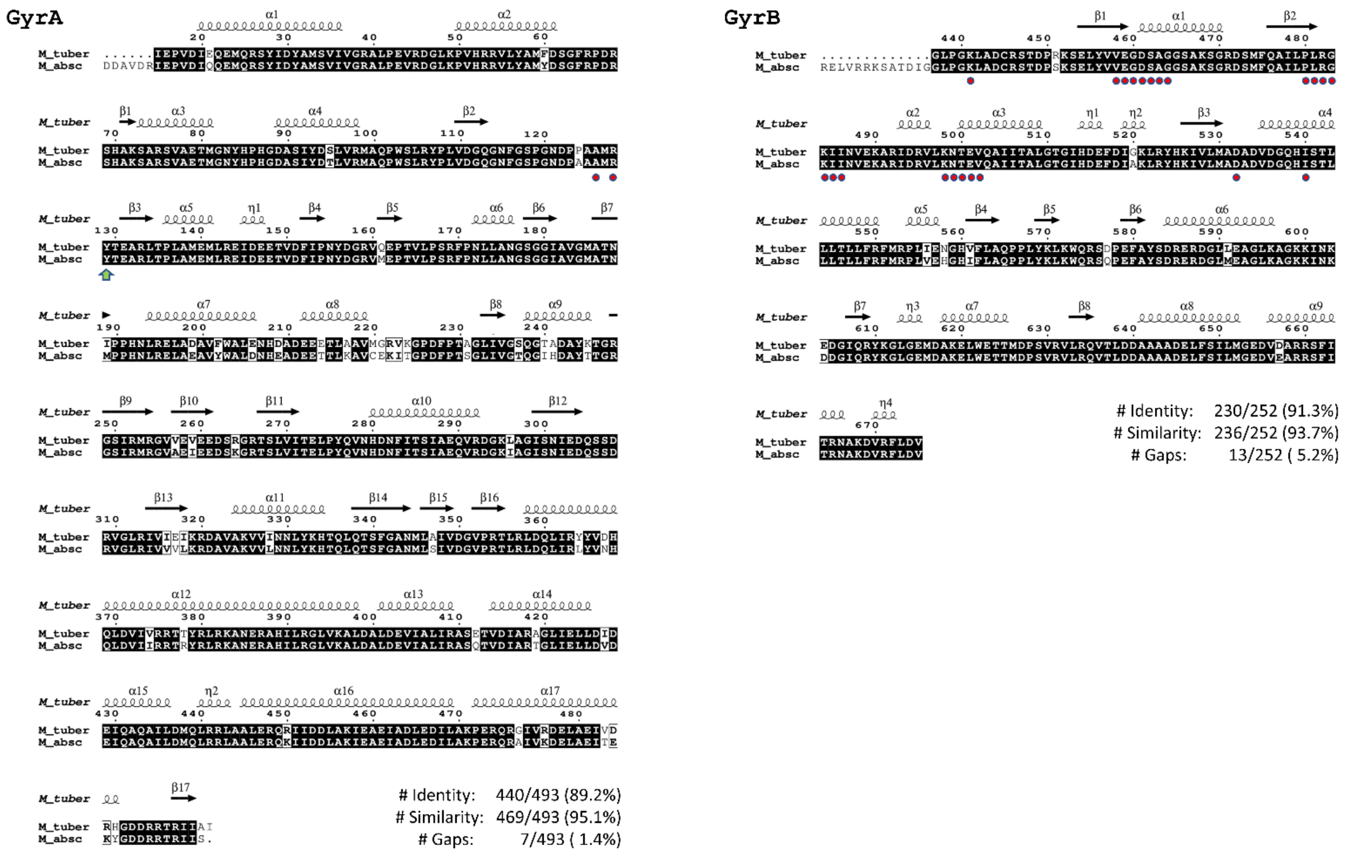

Figure S5. The schematic binding modes of docked FQs to the *M. abscessus* homology model.

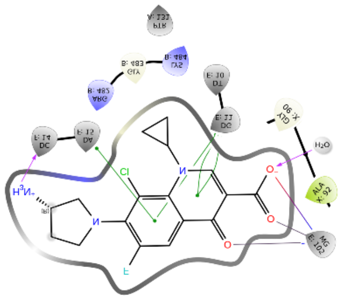

Clinafloxacin

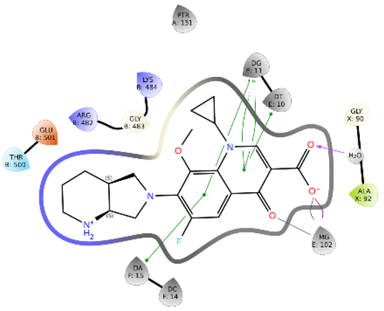

Moxifloxacin

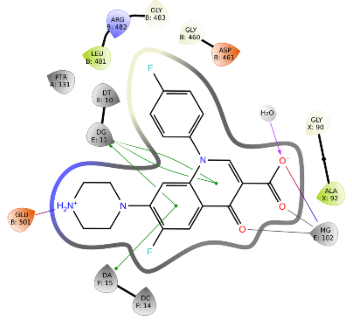

Sarafloxacin

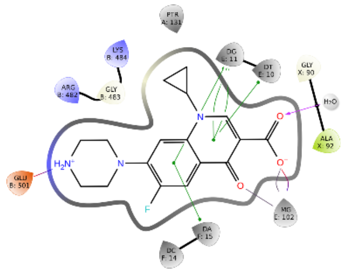

Ciprofloxacin

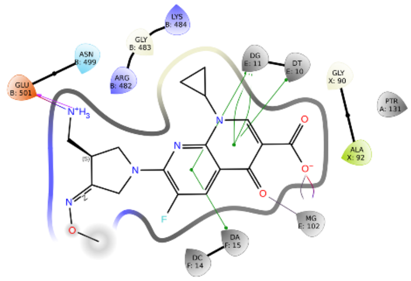

Gemifloxacin

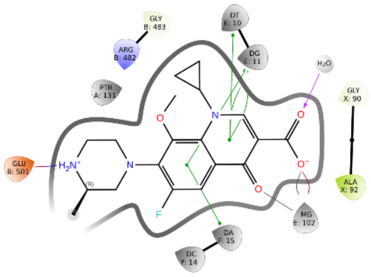

Gatifloxacin

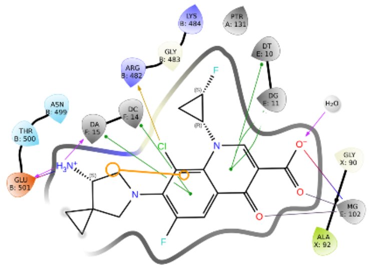

Sitafloxacin

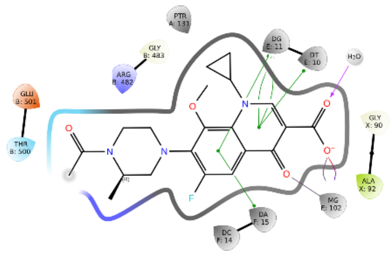

DHQ1

Figure S6. 3D binding site view of best docked compound – clinafloxacin to the *M. abscessus* homology model structure with PTR129.

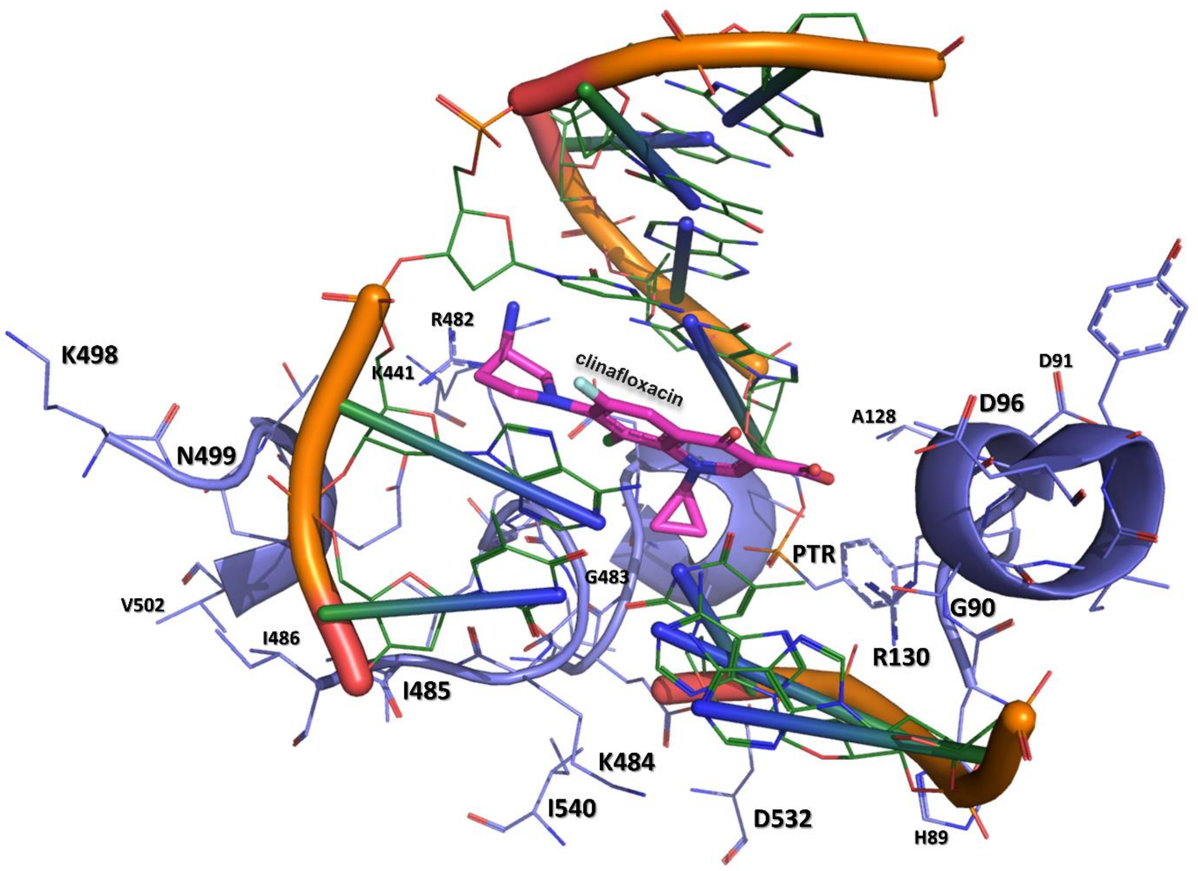

GOUET, P., ROBERT, X. & COURCELLE, E. 2003. ESPript/ENDscript: Extracting and rendering sequence and 3D information from atomic structures of proteins. *Nucleic Acids Res,* 31**,** 3320-3.
